# Lymphocyte-to-C-reactive protein ratio predicts prognosis in unresectable locally advanced non-small cell lung cancer patients

**DOI:** 10.1080/07853890.2025.2487629

**Published:** 2025-04-03

**Authors:** Yingying Xu, Jinping Li, Xiang Ji, Qingqing Chen, Zhengcao Liu, Shengjun Ji

**Affiliations:** ^a^ Department of Radiotherapy and Oncology, The Second Affiliated Hospital of Soochow University Suzhou, Suzhou, China; ^b^ Department of Gastroenterology, Fangzi People’s Hospital, Weifang, China; ^c^ Department of Radiotherapy and Oncology, The affiliated Suzhou Hospital of Nanjing Medical University, Gusu School, Nanjing Medical University, Suzhou, China

**Keywords:** LCR, LA-NSCLC, prognostic index, chemoradiotherapy

## Abstract

**Background:**

The lymphocyte-to-C-reactive protein ratio (LCR) is a promising inflammation-based tool for assessing the status of patients with malignant tumours. This study evaluated the ability of LCR to predict the prognosis of patients with unresectable locally advanced non-small cell lung cancer (LA-NSCLC) after chemoradiotherapy.

**Methods:**

We retrospectively investigated 206 consecutive patients with unresectable LA-NSCLC who underwent chemoradiotherapy between January 2016 and November 2019. The LCR was calculated from the differential count by dividing the absolute lymphocyte count by the C-reactive protein level. The optimal cut-off value of LCR was determined using the receiver operating characteristic (ROC) curve, and the enrolled patients were divided into two groups for further analysis according to LCR. Overall survival (OS) and disease-free survival (DFS) were assessed using univariate and multivariate Cox regression analyses.

**Results:**

In patients with unresectable LA-NSCLC, the level of LCR was significantly associated with pathology (*p* = 0.042) and TNM stage (*p* = 0.002). High LCR and low LCR patients had different distinct outcomes (median OS: 36 vs. 34 months, *p* < 0.0001) and recurrence risk (median DFS: 31 vs. 23 months, *p* < 0.001). Univariate analysis indicated that Eastern Cooperative Oncology Group (ECOG) performance status, TNM stage, CEA level, response, neutrophil-to-lymphocyte ratio (NLR), lymphocyte-to-monocyte ratio (LMR), systemic immune inflammation index (SII), and LCR were predictors of OS and DFS. Multivariate analysis showed that a high LCR was an independent prognostic factor for OS (hazard ratio [HR], 0.526; 95% CI, 0.364-0.762; *p* = 0.001) and DFS (HR, 0.390; 95% CI, 0.275-0.554; *p* < 0.001)

**Conclusion:**

LCR is a promising prognostic index in patients with LA-NSCLC undergoing chemoradiotherapy, and an increase in the LCR level contributes to better outcomes.

## Introduction

Lung cancer is one of the most commonly diagnosed malignancies and the leading cause of cancer-related deaths in men and women worldwide [1]. More than 1/3 of the patients with non-small cell lung cancer (NSCLC) present with locally advanced disease at the time of diagnosis [2]. Currently, chemoradiotherapy is the standard treatment modality for patients with potentially ‘unresectable’ locally advanced non-small cell lung cancer (LA-NSCLC) [3,4]. Recent advances have improved the long-term outcomes of this deadly disease. Many clinical trials on promising therapies, such as immunotherapy and targeted therapy, are being performed, and the early results are inspiring [5,6]. Despite achievements in multimodality therapies, the reported survival of LA-NSCLC is associated with poor long-term survival rates, ranging between 20% and 25% [7,8]. Recurrence is the main obstacle to long-term survival of patients with LA-NSCLC. Clinical studies on useful indices for predicting prognosis are being actively performed worldwide. Nevertheless, the complexity of the signal transmission mechanisms and heterogeneity of the tumour make it difficult to identify the prognosis risk for patients. The pursuit of indices that may expand prognostic risk predictions in LA-NSCLC is ongoing.

Systemic inflammation is recognized as a hallmark of the biological processes in many malignant cancers and is associated with a more aggressive disease course [9]. It can directly and indirectly upregulate the immune response *via* inflammatory and immune cytokines [10,11]. Clinical evidence has demonstrated that systemic inflammation is closely related to oncological outcomes in multiple types of malignancies, including NSCLC [12–16]. Recently, several inflammatory factors have been reported for NSCLC, such as neutrophils, monocytes, lymphocytes, platelets, C-reactive protein, and albumin [17–21]. In addition, some comprehensive inflammation-based indices, including the systemic immune inflammation index (SII), C-reactive protein-to-albumin ratio (CAR), neutrophil-to-lymphocyte ratio (NLR), platelet-to-lymphocyte ratio (PLR) and lymphocyte-to-monocyte ratio (LMR), have yielded more robust results than the single inflammatory index [22–26]. Recently, a novel multidimensional comprehensive inflammation-based index, the lymphocyte-C-reactive protein ratio (LCR), was established in gastric cancer by Okugawa et al.^’^s clinical research [27]. This comprehensive index has been reported in colorectal cancer, oesophageal cancer, hepatocellular carcinoma and oral cavity squamous cell carcinoma and is of great significance in terms of predicting prognosis [28–31]. However, the correlation between LCR and LA-NSCLC has not been elucidated.

The aim of this study was to investigate the relationship between the LCR and long-term outcomes of LA-NSCLC patients, as well as to compare to traditional inflammation-based indexes. In addition, we also tried to evaluate survival after chemoradiotherapy and to further investigate the survival difference based on stage IIIA and stage IIIB NSCLC.

## Materials and methods

### Patient selection

This retrospective study was approved by the hospital review board and was performed in accordance with the Declaration of Helsinki. Electronic medical data were extracted from 206 LA-NSCLC patients who underwent chemoradiotherapy between January 2016 and November 2019. The inclusion criteria in this study were as follows: (1) patients aged 18 years or older; (2) patients with histopathologically verified NSCLC; (3) patients who were diagnosed with stage IIIA or IIIB NSCLC according to the 7th edition of the American Joint Committee (AJCC) on cancer staging system [32]; (4) patients who consented and underwent chemoradiotherapy; (5) complete chemoradiotherapy information record; and (6) adequate data on overall survival (OS) and disease-free survival (DFS). The following exclusion criteria were applied: (1) the presence of other concurrent cancers, (2) patients receiving anticancer treatments before chemoradiotherapy, (3) evidence of infection or haematological disease, and (3) patients who were lost to follow-up.

### Therapeutic procedures

Radiotherapy was performed using a three-dimensional or intensity-modulated radiation therapy planning system. 6 MV X-rays were used for radiotherapy (2 Gy daily, total 60–66 Gy). We identified the primary tumour and involved lymph nodes using enhanced computed tomography (CT) or positron emission tomography-CT (PET-CT). The clinical target volume plus a 5–10 mm margin was defined as the planned target volume. The individualized radiotherapy plan for the patient was approved by two experienced radiotherapists. In addition to radiotherapy, patients with LA-NSCLC were administered chemotherapy drugs. Cisplatin*/*docetaxel, cisplatin/etoposide, cisplatin/vinorelbine, and cisplatin/paclitaxel were administered to the patients, and the platinum-based chemotherapeutic regimens were determined by the oncologists. The basic principle of therapy is that low-dose chemotherapy drugs and radiation should be administered to elderly patients with weak constitution.

### The evaluation of the efficacy for chemoradiotherapy

We used the Response Evaluation Criteria for Solid Tumours (RECIST version 1.1) to evaluate radiographic tumour responses, which were quantified as the best overall response and maximum tumour shrinkage [33]. The criteria were as follows: complete response (CR) was defined as the disappearance of all target lesions; partial response (PR) was defined as a decrease in the sum of the target lesion diameters by at least 30% compared to the baseline diameters; progressive disease (PD) was defined as an increase of at least 20% in the sum of the target lesion diameters compared to the smallest sum during the study; and stable disease (SD) was defined as insufficient shrinkage or expansion to qualify as PR or PD. Specialists in radiology and clinical oncology discussed the changes in target lesions and made decisions about efficacy evaluation.

### Ascertainment of LCR and other inflammation-related indexes

Laboratory blood sample data were obtained from all patients within seven days prior to chemoradiotherapy, including neutrophils, monocytes, lymphocytes, platelets, C-reactive protein (CRP), albumin, carcinoembryonic antigen (CEA), cytokeratin 19 fragments (CYFRA21-1) and squamous cell carcinoma antigen (SCC). The LCR was calculated according to the following formula: total lymphocyte (10^9^/L)/CRP (mg/L). The NLR was defined as the ratio of the absolute neutrophil count to the absolute lymphocyte count. The LMR was defined as the ratio of the absolute lymphocyte count to the absolute monocyte count. PLR is defined as the ratio of absolute platelet count to absolute lymphocyte count. The definitions of SII and prognostic nutritional index (PNI) were calculated as follows: SII = platelet count multiplied by neutrophil count and then divided by lymphocyte count; PNI = PNI = albumin level (g/L) + 5 × total lymphocyte count (10^9^/L).

### Outcome measurement

Patients were regularly followed up after chemoradiotherapy at three-month interval for the first year. Subsequently, routine follow-up was continued biannually for the next three years. Chest CT was performed at every follow-up visit, and imaging of other sites was performed according to the patient’s condition. The following data were extracted from the hospital electronic medical system: age at diagnosis, sex, Eastern Cooperative Oncology Group (ECOG) performance status, smoking history, pathology, differentiation grade, primary tumour location, tumour size, radiotherapy technique, treatment modality, TNM stage, survival months and survival status. In this study, the primary outcome measure was OS and the secondary outcome measure was DFS. OS was defined as the time from chemoradiotherapy to death from any cause or the date of the last follow-up. DFS was defined as the time from chemoradiotherapy until disease progression or death from any cause.

### Statistical analyses

All continuous variables are presented as categorical variables for better analysis. The chi-squared test was used to evaluate the association between LCR and categorical variables. Receiver operating characteristic (ROC) curves with Youden’s index were used to rank the different inflammatory-based indices according to their predictive capacity for outcome. The OS and DFS curves were generated using the Kaplan-Meier method, and statistical differences were determined using the log-rank method. The Cox proportional hazards model was used for univariate and multivariate analyses, and the hazard ratios (HR) for recurrence and death were determined. Statistical differences with a two-sided *P*-value of <0.05 were regarded as significant. The aforementioned statistical calculations were performed using SPSS software (version 20.0; SPSS, Chicago, IL, USA) and GraphPad Prism8 software (CA, USA).

## Results

### Patients characteristics

Clinicopathological characteristics of the patients included in this study are shown in Table 1. The sex ratio of the enrolled patients was 100(48.5%) males and 106(51.5%) females. The median age of the patients was 58.5 years (interquartile range [IQR] 51.0–66.25), and 99(48.1%) patients had a smoking history. Histological subtypes identified 117(56.8%) patients with adenocarcinoma and 89(43.2%) with squamous cell carcinoma. More than half (120/206, 58.3%) were stage III B, and the others (86/206, 41.7%) had stage IIIA disease. The majority of patients (*n* = 126, 61.2%) received a total radiation dose of 60 Gy, and 80 patients (38.8%) received more than 60 Gy. A total of 54 patients received docetaxel-cisplatin chemotherapy, 27 received etoposide-cisplatin chemotherapy, 36 received vinorelbine-cisplatin chemotherapy, and 89 received paclitaxel-cisplatin chemotherapy.

**Table 1.** Clinicopathological variables in LA-NSCLC patients.

Variable	*N* (%)
Age (years)	
<60	122 (59.2)
≥60	84 (40.8)
Gender	
Female	106 (51.5)
Male	100 (48.5)
ECOG performance status	
0	162 (78.6)
1	44 (21.4)
Smoking history	
Never	107 (51.9)
Current or former	99 (48.1)
Pathology	
AD	117 (56.8)
SCC	89 (43.2)
Differentiation grade	
Well	87 (42.2)
Moderate/Poor	119 (57.8)
Tumour location	
Upper lobes	105 (51.0)
Lower/middle lobes	101 (49.0)
Tumour size	
≤4 cm	70 (34.0)
>4 cm	136 (66.0)
Radiotherapy technique	
IMRT	105 (51.0)
3D-CRT	101 (49.0)
Treatment modality	
SCRT	99 (48.1)
CCRT	107 (51.9)
TNM stage	
IIIA	86 (41.7)
IIIB	120 (58.3)
CEA	
Normal	82 (39.8)
High	124 (60.2)
CYFRA21-1(ng/mL)	
Normal	112 (54.4)
High	94 (45.6)
SCC	
Normal	93 (45.1)
High	113 (54.9)
Response	
SD+PD	126 (61.2)
CR+PR	80 (38.8)
LCR	
Low	82 (39.8)
High	124 (60.2)
NLR	
Low	145 (70.4)
High	61 (29.6)
PLR	
Low	124 (60.2)
High	82 (39.8)
LMR	
Low	198 (96.1)
High	8 (3.9)
SII	
Low	141 (68.4)
High	65 (31.6)
PNI	
Low	147 (71.4)
High	59 (28.6)

ECOG: Eastern Cooperative Oncology Group; AD: adenocarcinoma; SCC: squamous cell carcinoma; IMRT: intensity-modulated radiation therapy; 3D-CRT: three-dimensional conformal radiation therapy; SCRT: sequential chemoradiotherapy; CCRT: concurrent chemoradiotherapy; CEA: arcinoembryonic antigen; CYFRA21-1: cytokeratin 19 fragments; SCC: squamous cell carcinoma antigen; CR: complete response; PR: partial response; SD: Stable disease; PD: Progressive disease; LCR: lymphocyte-C-reactive protein ratio; NLR: neutrophil-to-lymphocyte ratio; PLR: platelet-to-lymphocyte ratio; LMR: lymphocyte-to-monocyte ratio; SII: systemic immune-inflammation index; PNI: prognostic nutritional index.

### Correlation between LCR and patient characteristics

Next, we evaluated the correlation between LCR and clinicopathological variables in patients with LA-NSCLC (Table 2). A total of 124 (60.2%) patients were allocated to the high LCR group (≥0.21) and 82 (39.8%) patients were allocated to the low LCR group (<0.21). A higher level of LCR was significantly associated with adenocarcinoma (*p* = 0.042) and stage IIIB (*p* = 0.002) LA-NSCLC. No significant differences were observed in age (*p* = 0.575), sex (*p* = 0.181), ECOG performance status (*p* = 0.166), smoking history (*p* = 0.266), differentiation grade (*p* = 0.802), primary tumour location (*p* = 0.441), tumour size (*p* = 0.312), radiotherapy technique (*p* = 0.933), and treatment modality (*p* = 0.796).

**Table 2.** Association of the lymphocyte-to-C-reactive protein ratio (LCR) with the clinicopathological variables.

	LCR		
Variable	Low LCR (*n* = 82)	High LCR (*n* = 124)	*P* value
Age (years)			0.575
<60	51 (62.2)	71 (57.3)	
≥60	31 (37.8)	53 (42.7)	
Gender			0.181
Female	37 (45.1)	69 (55.6)	
Male	45 (54.9)	55 (44.4)	
ECOG performance status			0.166
0	60 (73.2)	102 (82.3)	
1	22 (26.8)	22 (17.7)	
Smoking history			0.266
Never	47 (57.3)	60 (48.4)	
Current or former	35 (42.7)	64 (51.6)	
Pathology			0.042
AD	39 (47.6)	78 (62.9)	
SCC	43 (52.4)	46 (37.1)	
Differentiation grade			0.802
Well	36 (43.9)	51 (41.1)	
Moderate/Poor	46 (56.1)	73 (58.9)	
Tumour location			0.441
Upper lobes	45 (54.9)	60 (48.4)	
Lower/middle lobes	37 (45.1)	64 (51.6)	
Tumour size			0.312
≤4 cm	24 (29.3)	46 (37.1)	
>4 cm	58 (70.7)	78 (62.9)	
Radiotherapy technique			0.933
IMRT	41 (50.0)	64 (51.6)	
3D-CRT	41 (50.0)	60 (48.4)	
Treatment modality			0.796
SCRT	38 (46.3)	61 (49.2)	
CCRT	44 (53.7)	63 (50.8)	
TNM stage			0.002
IIIA	23 (28.0)	63 (50.8)	
IIIB	59 (72.0)	61 (49.2)	
CEA			0.803
Normal	34 (41.5)	48 (38.7)	
High	48 (58.5)	76 (61.3)	
CYFRA21-1(ng/mL)			0.160
Normal	50 (61.0)	62 (50.0)	
High	32 (39.0)	62 (50.0)	
SCC			0.478
Normal	40 (48.8)	53 (42.7)	
High	42 (51.2)	71 (57.3)	
Response			0.999
SD+PD	50 (61.0)	76 (61.3)	
CR+PR	32 (39.0)	48 (38.7)	

ECOG: Eastern Cooperative Oncology Group; AD: adenocarcinoma; SCC: squamous cell carcinoma; IMRT: intensity-modulated radiation therapy; 3D-CRT: three-dimensional conformal radiation therapy; SCRT: sequential chemoradiotherapy; CCRT: concurrent chemoradiotherapy; CEA: arcinoembryonic antigen; CYFRA21-1: cytokeratin 19 fragments; SCC: squamous cell carcinoma antigen; CR: complete response; PR: partial response; SD: Stable disease; PD: Progressive disease.

### Comparison of the LCR and other inflammation-related markers

**Figure 1.**
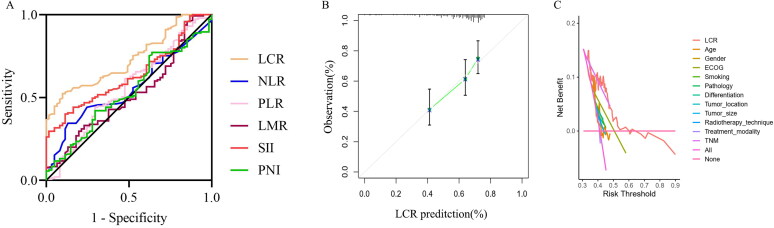
Comparison of predictive performances of LCR and other known biomarkers by receiver operating characteristic (ROC) curves (a), calibration curve (B) and decision curve analysis (C). Abbreviations: LCR, lymphocyte-C-reactive protein ratio; NLR, neutrophil-to-lymphocyte ratio; PLR, platelet-to-lymphocyte ratio; LMR, lymphocyte-to-monocyte ratio; SII, systemic immune-inflammation index, PNI, prognostic nutritional index.

The median LCR was 0.190 (IQR, 0.147–0.248). In addition, the median level of NLR, PLR, LMR, SII and PNI were 2.800 (IQR, 1.875–3.800), 135.250 (IQR, 106.875–175.900), 2.663 (IQR, 1.315–3.857), 562.150 (IQR, 372.525–710.200), and 50.825 (IQR, 48.088–54.163), respectively. Table 3 summarizes the features of the LCR and other inflammation-related indices. Next, we used the ROC with Youden’s index to determine which inflammatory-based indices were the best for predicting outcomes. ROC analysis of the recurrence status showed that the optimal cutoff values of the LCR, NLR, PLR, LMR, SII and PNI were 0.21 (area under the curve [AUC], 0.731; *p* < 0.0001), 3.55 (AUC, 0.556; *p* = 0. 203), 154.85 (AUC, 0.557; *p* = 0.208), 0.55 (AUC, 0.515; *p* = 0.729), 662.45 (AUC, 0.633; *p* = 0.003), and 48.50 (AUC, 0.530; *p* = 0.498), respectively. Among these prognostic indexes, we identified that the LCR consistently had a higher AUC value than the other inflammation-related indexes (AUC: 0.515–0.633) for LA-NSCLC (Figure 1(A)). The calibration plot was also drawn and exhibited an acceptable accuracy for prediction (Figure 1(B)). In addition, Decision curve analysis (DCA) confirmed that LCR had the highest net benefit, suggesting that LCR can be effectively applied for clinical prediction (Figure 1(C)).

**Table 3.** Comparison of the area under the curve values of inflammation-related prognostic scoring systems.

Variables	Median (IQR)	AUC	95% CI	*P* value
LCR	0.190 (0.147–0.248)	0.731	0.663–0.799	<0.001
NLR	2.800 (1.875–3.800)	0.556	0.476–0.637	0.203
PLR	135.250 (106.875–175.900)	0.557	0.468–0.643	0.208
LMR	2.663 (1.315–3.857)	0.515	0.429–0.601	0.729
SII	562.150 (372.525–710.200)	0.633	0.557–0.710	0.003
PNI	50.825 (48.088–54.163)	0.530	0.446–0.614	0.498

IQR: interquartile range; LCR: lymphocyte-C-reactive protein ratio; NLR: neutrophil-to-lymphocyte ratio; PLR: platelet-to-lymphocyte ratio; LMR: lymphocyte-to-monocyte ratio; SII: systemic immune-inflammation index; PNI: prognostic nutritional index.

### Outcomes

**Figure 2.**
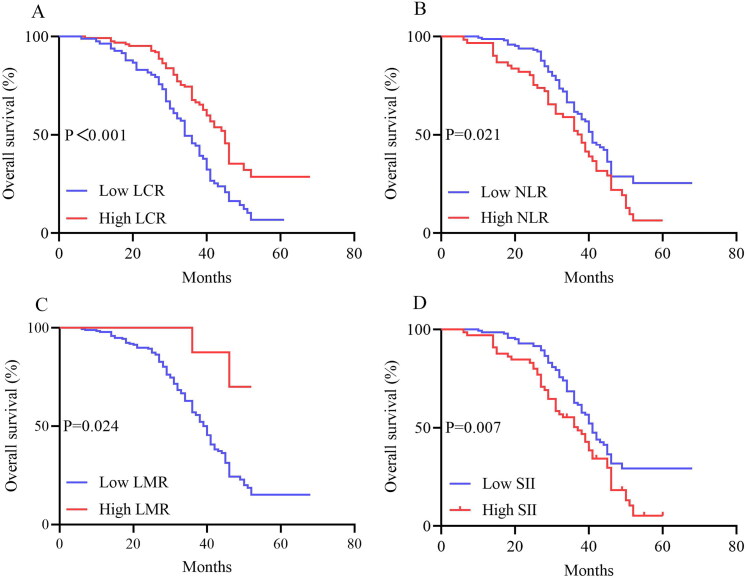
Kaplan–Meier Curves for OS between the high LCR group (≥0.21) and low LCR group (<0.21) (A), high NLR group and low NLR group (B), high LMR group and low LMR group (C), high SII group and low SII group (D) in LA-NSCLC patients. Abbreviations: OS, overall survival; LCR, lymphocyte-C-reactive protein ratio; NLR, neutrophil-to-lymphocyte ratio; LMR, lymphocyte-to-monocyte ratio; SII, systemic immune-inflammation index; LA-NSCLC, locally advanced non-small cell lung cancer.

**Figure 3.**
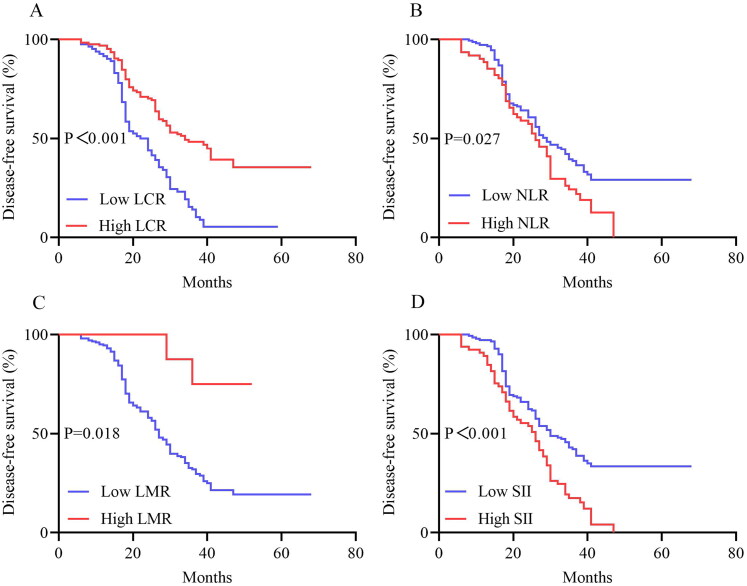
Kaplan–Meier Curves for DFS between the high LCR group (≥0.21) and low LCR group (<0.21) (A), high NLR group and low NLR group (B), high LMR group and low LMR group (C), high SII group and low SII group (D) in LA-NSCLC patients. Abbreviations: DFS, disease-free survival; LCR, lymphocyte-C-reactive protein ratio; NLR, neutrophil-to-lymphocyte ratio; LMR, lymphocyte-to-monocyte ratio; SII, systemic immune-inflammation index; LA-NSCLC, locally advanced non-small cell lung cancer.

**Figure 4.**
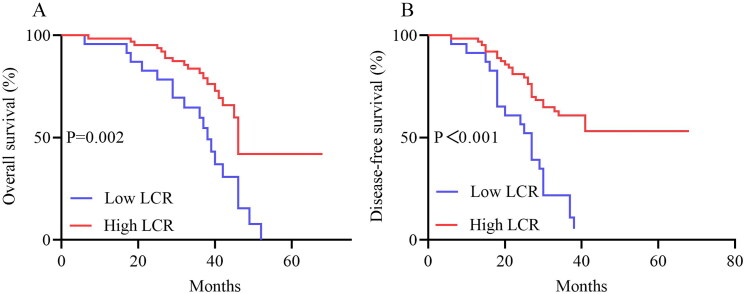
OS (A) And DFS (B) of patients with high and low LCR in stage IIIA with LA-NSCLC. Abbreviations: OS, overall survival; DFS, disease-free survival; LCR, lymphocyte-C-reactive protein ratio; LA-NSCLC, locally advanced non-small cell lung cancer.

**Figure 5.**
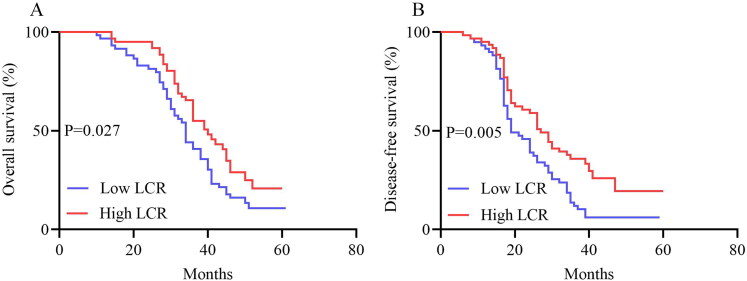
OS (A) And DFS (B) of patients with high and low LCR in stage IIIB with LA-NSCLC. Abbreviations: OS, overall survival; DFS, disease-free survival; LCR, lymphocyte-C-reactive protein ratio; LA-NSCLC, locally advanced non-small cell lung cancer.

The median follow-up time was 37 months (IQR: 19–51 months). Compared with patients who had high LCR, the median OS and DFS of patients who had low LCR were significantly shortened (36 vs. 34 months, *p* < 0.001; 31 vs. 23 months, *p* < 0.001). The 82 patients with low LCR had a significantly lower OS (*p* < 0.001) and DFS (*p* < 0.001) than the 124 patients with high LCR. In addition, OS and DFS analyses showed that patients with a high NLR, low LMR, and high SII had poorer survival benefits than those with a low NLR, high LMR, and low SII after chemoradiotherapy (Figures 2, 3). Further analyses based on TNM stage were performed. A total of 86 patients were in the stage IIIA subgroup and 120 patients in the stage IIIB subgroup. The results presented in Figure 4 show the OS and DFS curve features according to the LCR in the stage IIIA subgroups. The high LCR group had significantly better OS and DFS than the low LCR group (*p* = 0.002 and *p* < 0.0001, respectively). Similar results were observed in patients with stage IIIB disease (OS, *p* = 0.027; DFS, *p* = 0.005, Figure 5).

### Predictive value of LCR

Using the univariate Cox model, ECOG performance status (*p* = 0.001), TNM stage (*p* = 0.005), CEA level (*p* = 0.008), response (*p* = 0.009), NLR (*p* = 0.021), LMR (*p* = 0.024), SII (*p* = 0.007), and LCR (*p* < 0.001) were identified as significant determinants of OS. In addition, a 1 score of for ECOG performance status (*p* < 0.001), stage IIIB (*p* < 0.001), high CEA (*p* = 0.017), worse response (*p* = 0.017), high NLR (*p* = 0.027), low LMR (*p* = 0.018), high SII (*p* < 0.001), and low LCR (*p* < 0.001) were associated with lower DFS (Tables 4, 5). Multivariable Cox regression analysis indicated that only LCR was an independent predictor of OS (hazard ratio [HR] 1.42, 95% CI 0.364–0.762, *p* = 0.001) (Table 6). In the DFS multivariable Cox regression analysis, we found similar results for LCR (HR 0.390, 95% CI 0.275–0.554; *p* < 0.001). We also found that TNM stage (HR 1.527, 95% CI 1.062–2.196, *p* = 0.022) and SII (HR 1.937, 95% CI 1.234–3.040, *p* = 0.004) were independent predictors of DFS.

**Table 4.** Univariate analyses of clinicopathological variables for OS and DFS in LA-NSCLC patients.

	OS	DFS				
Variables	HR	95% CI	*P* value	HR	95% CI	*P* value
Age (years)			0.263			0.130
<60	Reference			Reference		
≥60	1.220	0.861–1.730		1.288	0.929–1.786	
Gender			0.710			0.333
Female	Reference			Reference		
Male	1.068	0.755–1.512		1.175	0.848–1.624	
ECOG performance status			0.001			<0.001
0	Reference			Reference		
1	1.888	1.294–2.753		1.986	1.382–2.853	
Smoking history			0.171			0.398
Never	Reference			Reference		
Current or former	1.275	0.900–1.806		1.151	1.831–1.595	
Pathology			0.392			0.085
AD	Reference			Reference		
SCC	1.165	0.822–1.650		1.333	0.961–1.849	
Differentiation grade			0.369			0.742
Well	Reference			Reference		
Moderate/Poor	1.174	0.827–1.667		1.057	0.761–1.468	
Tumor location			0.697			0.614
Upper lobes	Reference			Reference		
Lower/middle lobes	0.933	0.659–1.321		1.088	0.784–1.509	
Tumor size			0.349			0.989
≤4 cm	Reference			Reference		
>4 cm	0.841	0.585–1.209		1.003	0.714–1.415	
Radiotherapy technique			0.263			0.816
IMRT	Reference			Reference		
3D-CRT	0.819	0.578–1.162		0.962	0.694–1.333	
Treatment modality						
SCRT	Reference		0.853	Reference		
CCRT	1.034	0.729–1.465		1.161	0.836–1.614	0.373
TNM stage			0.005			<0.001
IIIA	Reference			Reference		
IIIB	1.726	1.181–2.523		1.949	1.375–2.762	
CEA			0.008			0.017
Normal	Reference			Reference		
High	0.616	0.431–0.881		0.663	0.473–0.928	
CYFRA21-1(ng/mL)			0.115			0.245
Normal	Reference			Reference		
High	1.324	0.934–1.786		1.214	0.876–1.682	
SCC			0.376			0.815
Normal	Reference			Reference		
High	0.854	0.603–1.210		0.961	0.692–1.335	
Response			0.00			0.017
SD+PD	Reference		9	Reference		
CR+PR	0.618	0.432–0.885		0.662	0.472–0.928	

OS: overall survival; DFS: disease-free survival; LA-NSCLC: locally advanced non-small cell lung cancer; ECOG: Eastern Cooperative Oncology Group; AD: adenocarcinoma; SCC: squamous cell carcinoma; IMRT: intensity-modulated radiation therapy; 3D-CRT: three-dimensional conformal radiation therapy; SCRT: sequential chemoradiotherapy; CCRT: concurrent chemoradiotherapy; CEA: arcinoembryonic antigen; CYFRA21-1: cytokeratin 19 fragments; SCC: squamous cell carcinoma antigen; CR: complete response; PR: partial response; SD: Stable disease; PD: Progressive disease.

**Table 5.** Univariate analyses of inflammation-related prognostic scoring systems for OS and DFS in LA-NSCLC patients.

	OS			DFS		
Variables	HR	95% CI	*P* value	HR	95% CI	*P* value
LCR			<0.001			<0.001
Low	Reference			Reference		
High	0.487	0.344–0.690		0.403	0.289–0.560	
NLR			0.021			0.027
Low	Reference			Reference		
High	1.529	1.067–2.192		1.477	1.046–2.084	
PLR			0.155			0.101
Low	Reference			Reference		
High	1.288	0.909–1.825		1.315	0.948–1.826	
LMR			0.024			0.018
Low	Reference			Reference		
High	0.103	0.014–0.738		0.093	0.013–0.663	
SII			0.007			<0.001
Low	Reference			Reference		
High	1.633	1.147–2.326		1.935	1.384–2.705	
PNI			0.276			0.180
Low	Reference			Reference		
High	0.798	0.532–1.197		0.772	0.530–1.126	

OS: overall survival; DFS: disease-free survival; LA-NSCLC: locally advanced non-small cell lung cancer; LCR: lymphocyte-C-reactive protein ratio; NLR: neutrophil-to-lymphocyte ratio; PLR: platelet-to-lymphocyte ratio; LMR: lymphocyte-to-monocyte ratio; SII: systemic immune-inflammation index; PNI: prognostic nutritional index.

**Table 6.** Multivariate analyses of prognostic factors for OS and DFS in LA-NSCLC patients.

	OS			DFS		
Variables	HR	95% CI	*P* value	HR	95% CI	*P* value
ECOG performance status			0.111			0.065
0	Reference			Reference		
1	1.393	0.927–2.092		1.437	0.977–2.112	
TNM stage			0.059			0.022
IIIA	Reference			Reference		
IIIB	1.459	0.985–2.159		1.527	1.062–2.196	
CEA			0.517			0.194
Normal	Reference			Reference		
High	1.613	0.380–6.853		2.608	0.614–11.066	
Response			0.249			0.072
SD+PD	Reference			Reference		
CR+PR	0.425	0.099–1.822		0.262	0.061–1.129	
LCR			0.001			<0.001
Low	Reference			Reference		
High	0.526	0.364–0.762		0.390	0.275–0.554	
NLR			0.302			0.549
Low	Reference			Reference		
High	1.292	0.794–2.101		1.151	0.726–1.826	
LMR			0.100			0.076
Low	Reference			Reference		
High	0.190	0.026–1.378		0.166	0.023–1.203	
SII			0.174			0.004
Low	Reference			Reference		
High	1.398	0.862–2.265		1.937	1.234–3.040	

OS: overall survival; DFS: disease-free survival; LA-NSCLC: locally advanced non-small cell lung cancer; ECOG: Eastern Cooperative Oncology Group; CEA: arcinoembryonic antigen; CR: complete response; PR: partial response; SD: Stable disease; PD: Progressive disease; LCR: lymphocyte-C-reactive protein ratio; NLR: neutrophil-to-lymphocyte ratio; LMR: lymphocyte-to-monocyte ratio; SII: systemic immune-inflammation index.

## Discussion

Mounting evidence indicates that the systemic inflammatory response plays an important role in cancer survival, but the components of the systemic inflammatory environment that accurately predict long-term outcomes in LA-NSCLC patients remain unclear. This study is the first to validate that LCR is an independent marker of prognosis in LA-NSCLC patients and is superior to the NLR, PLR, LMR, SII, and PNI scores in terms of predicting prognosis.

In recent years, the diagnosis, therapy, and monitoring of malignant tumors have been affected, and the ability to predict long-term outcomes in LA-NSCLC is unsatisfactory. Although the TNM staging system acts as a common prognostic evaluation system, its ability to distinguish between high and low risk for prognosis has been questioned [34,35]. Unfortunately, the long-term outcomes for NSCLC patients are discrepant, even in the same TNM stage. The lack of adequate understanding of the biological characteristics may be the main reason for this disappointing result. Therefore, biological biomarkers other than the TNM staging system need to be further verified. It is generally acknowledged that inflammation plays an important role in the proliferation, metastasis, and immune escape of tumours [36–38]. Moreover, systemic inflammatory response markers have been proven to be related to the prognosis of NSCLC patients in prior studies and can be independent prognostic factors [25,39]. Peripheral blood cells such as neutrophils, lymphocytes, monocytes, and platelets are regarded as systemic inflammatory cells. Nevertheless, previous studies have mainly focused on their combinations, including the NLR, PLR, LMR, and SII [40,41]. LCR has recently been recognized as a better predictor of long-term outcomes in solid tumours [30,31]. However, the predictive value of LCR in LA-NSCLC patients remains unclear. We conducted this study to assess the prognostic significance of systemic inflammatory response markers and to determine the best marker for improving prognostic evaluation in LA-NSCLC patients.

Okugawa et al. were the first to evaluate the prognostic value of LCR in patients with gastric cancer [27]. In terms of long-term outcomes, the results showed that low LCR was an independent prognostic factor for OS and DFS. Furthermore, they verified the prognostic impact of LCR in metastatic and nonmetastatic GC cancers. Lu et al. ‘s study involving a multicentre cohort indicated that the LCR score differentiated two groups of intrahepatic cholangiocarcinoma patients with distinct prognoses [42]. In the primary and validation cohorts, they found that the LCR score remained a significant and independent predictor in multivariate analyses. Moreover, the LCR score had the most ­reliable predictive ability compared to other inflammatory-based scores. Zhang et al. reported that bladder cancer patients with a high LCR tended to have a better prognosis and functioned as a prognostic marker for OS and DFS [43]. In addition, LCR has also been identified as a reliable prognostic marker in patients with hepatocellular carcinoma and gastric cancer based on the retrospective studies of Zhang et al. [44] and Miyatani et al. [45]. Hwang et al. reported the clinical significance of the C-reactive protein-to-lymphocyte count ratio in NSCLC patients undergoing curative surgical resection [46]. However, the included patients were treated with surgery rather chemoradiotherapy. Furthermore, prognostic evaluation of locally advanced disease has not been conducted. In summary, these findings provide more theoretical evidence for the prognostic value of LCR in patients with solid tumours.

In this retrospective study, we used the ROC curve to determine the optimal cutoff value for LCR. The results showed that LCR was significantly related to the pathology (*p* = 0.042) and TNM stage (*p* = 0.002). Meanwhile, patients with LA-NSCLC with a high LCR than the optimal cut-off value (>0.21) had a better OS and DFS compared with patients in the low LCR group. More importantly, based on stage stratification, we found that LCR was an independent prognostic factor for OS and DFS in patients with stage IIIA or IIIB disease. Univariate analysis for OS and DFS indicated that ECOG performance status, TNM stage, CEA, response, NLR, LMR, SII, and LCR were significantly related to OS and DFS, while only LCR was identified as an independent prognostic factor for OS and DFS in multivariate analysis. Furthermore, our study indicated that the presence of LCR was the optimal index for evaluating long-term outcomes in LA-NSCLC patients compared with other systemic inflammatory response indices. Therefore, our findings could shed light on the correlation between LCR and survival outcomes, aiming to provide in-depth prognostic details for LA-NSCLC patients following chemoradiotherapy.

The combination of lymphocytes and C-reactive protein levels may comprehensively reflect the association between decreased LCR and worse long-term outcomes. Lymphocytes represent the immune status of the host and are vital antitumor immune cells (especially B and T lymphocytes) [47–49]. Lymphocytes secrete cytokines (tumour necrosis factor-α and interferon-γ) to trigger an anti-tumor immune response, thereby inhibiting tumour growth [38,50]. Decreased lymphocyte levels may indicate an inactivated cellular immune response and high activation of the inflammatory response [51]. Furthermore, lymphocytes can infiltrate tumours and are related to better long-term outcomes in patients with malignant tumours, as these immune responses can inhibit tumour invasion and metastasis [52,53]. In contrast, serum C-reactive protein is a well-established systemic inflammatory index that promotes tumour angiogenesis by activating hypoxia-inducible factor-1a in the microenvironment [54]. Notably, accumulating evidence has demonstrated that increased C-reactive levels are associated with tumour progression, which can lead to poor prognosis [55,56]. Considering this evidence with biological mechanisms, LCR may adequately represent the characteristics of immunological and systemic inflammatory responses, where low LCR indicates a high risk of poor oncological long-term outcomes. The LCR score measurement provides the benefits of being entirely objective and simple to utilize for risk stratification during the regular evaluation of LA-NSCLC patients.

Our study had several limitations. First, it was a retrospective and single-centre cohort study with inherent defects, and selection bias was inevitable and may have affected the results of the study. Second, the optimal cut-off value for LCR and other inflammation-related indices was not consensual, and prospective studies are needed to validate these cut-off values. Third, the molecular biological mechanisms of lymphocytes and C-reactive protein in immune and inflammatory responses are still far from complete. Further research is needed to confirm these mechanisms. Finally, the intervention treatment of LA-NSCLC patients who experienced recurrence is inconsistent, which may influence the accuracy of LCR in evaluating prognosis.

## Conclusion

In summary, our study confirmed that LCR has a better predictive value for long-term outcomes in patients with LA-NSCLC than other inflammation-related indexes. Our study contributes to a comprehensive understanding of LCR and the immunological and systemic inflammatory responses in patients with LA-NSCLC. LCR offers the advantage of being easy to use for identifying high-risk or low-risk poor prognosis in the prognostic evaluation of LA-NSCLC patients.

## Supplementary Material

Title page.docx

## Data Availability

The datasets used and analysed during the current study are available from the corresponding author on reasonable request.
