# Supplementary material for: Lymphocyte-to-C-reactive protein ratio predicts prognosis in unresectable locally advanced non-small cell lung cancer patients
Source: Ann Med. 2025 Apr 3;57(1):2487629. doi: 10.1080/07853890.2025.2487629 (PMC11980205; doi:10.1080/07853890.2025.2487629)
Supplement: Title page.docx [file IANN_A_2487629_SM4342.docx]

**Title “Lymphocyte-to-C‑reactive protein ratio predicts prognosis in locally advanced non-small cell lung cancer patients *”***

| **Author name** | **E-mail** | **Institution** | **Telephone** | **Facsimile** |
| --- | --- | --- | --- | --- |
| Yingying Xu (first author) | 18913134622@163.com | Department of Radiotherapy & Oncology, The Second Affiliated Hospital of Soochow University Suzhou |  |  |
| Jinping Li | jinpingli1987@126.com | Department of Gastroenterology, Fangzi People’s Hospital |  |  |
| Xiang Ji | 397558317@qq.com | Department of Gastroenterology, Fangzi People’s Hospital |  |  |
| Qingqing Chen | chenqingqing0827@163.com | Department of Radiotherapy & Oncology, The affiliated Suzhou Hospital of Nanjing Medical University, Gusu School, Nanjing Medical University |  |  |
| Zhengcao Liu | zhengcaoliu@163.com | Department of Radiotherapy & Oncology, The affiliated Suzhou Hospital of Nanjing Medical University, Gusu School, Nanjing Medical University |  |  |
| Shengjun Ji( Corresponding author) | drshengjunji@163.com | Department of Radiotherapy & Oncology, The affiliated Suzhou Hospital of Nanjing Medical University, Gusu School, Nanjing Medical University |  |  |

**Affiliation:** **1** Department of Radiotherapy & Oncology, The Second Affiliated Hospital of Soochow University Suzhou, Suzhou, China **2** Department of Radiotherapy & Oncology, The affiliated Suzhou Hospital of Nanjing Medical University, Gusu School, Nanjing Medical University, Suzhou, China. **3** Department of Gastroenterology, Fangzi People’s Hospital, Weifang, China.
